# Supplementary material for: Rare-event sampling analysis uncovers the fitness landscape of the genetic code
Source: PLoS Comput Biol. 2023 Apr 17;19(4):e1011034. doi: 10.1371/journal.pcbi.1011034 (PMC10138212; doi:10.1371/journal.pcbi.1011034)
Supplement: S1 Text — (PDF) [file pcbi.1011034.s001.pdf]

S1 Text for
Rare-Event Sampling Analysis Uncovers the Fitness Landscape of the Genetic Code

Yuji Omachi

Graduate School of Sciences, The University of Tokyo, 7-3-1 Hongo, Tokyo, Japan.

Nen Saito*

Graduate School of Integrated Sciences for Life, Hiroshima University, Higashi-Hiroshima City, Hiroshima, Japan.
Exploratory Research Center on Life and Living Systems,
National Institutes of Natural Sciences, Okazaki, Aichi, Japan.
Universal Biology Institute, The University of Tokyo, Hongo, Tokyo, Japan.

Chikara Furusawa†

Graduate School of Sciences The University of Tokyo, Hongo, Tokyo, Japan.
Center for Biosystems Dynamics Research, RIKEN, Suita, Osaka, Japan.
Universal Biology Institute, University of Tokyo, Hongo, Tokyo, Japan.

(Dated: April 1, 2023)

* nensaito@hiroshima-u.ac.jp

† chikara.furusawa@riken.jp

I. RELAXING THE REQUIREMENT TO HAVE AT LEAST TWO INSTANCES OF ASP AND GLU

Relaxing the conditions for the ensemble that we have investigated (two Asp and Glu must be included), we also examined ensembles of the genetic code that contain 20 different amino acids (i.e., at least one Asp and Glu should be contained), and confirmed that the four cluster structure that is similar to Fig 2A was again obtained (S5 Fig; C-H). The code composition in each cluster (S5 Fig; C-D and C'-D') is similar to its counterpart in Fig 2C-D and C'-D': a code in the cluster C contains high polar requirement amino acids in the first and second column, a code in cluster D (orange cluster in Fig 2D and S5 Fig A) has those in the fourth column, in clusters E and F, those are located at the bottom half and upper half of the third column, respectively. However, the major difference is the number of high polar requirement amino acids (i.e., Asp and Glu): at the low-cost region with cost values comparative to SGC, almost all ($\sim 99\%$) codes in the ensemble contain only a single Asp and Glu (S8 Fig; A). The number of the codon coding Asp and Glu in SGC (i.e., two of each) is highly deviated (much more than 3-σ) from the average number ($\simeq 1$) in the distribution sampled from random code ensemble (S8 Fig; B), whereas the numbers of the codon for the other amino acids in SGC are within 3-σ in the distribution from random code ensemble. This may suggest that, apart from cost defined Eq.(1), the numbers of Asp and Glu have also undergone selection in the actual evolution of genetic code, and that these numbers have evolved to maintain being two. For an ensemble in which at least two Asp and Glu should be contained, the number of the codon for each amino acid falls within 2-σ of the distribution of the random code ensemble (S8 Fig; C), which is the reason we adopted this ensemble for our main analysis.

II. EXAMINING THE DIFFERENT COST FUNCTIONS THAT INCORPORATE AMINO ACID FREQUENCIES

To demonstrate generality of our conclusion, we additionally performed simulations with two different cost (fitness) functions: (1) the cost including amino acid frequencies in genome in extant organisms that mimicks the frequencies in the proteomes, and (2) the cost incorporating an empirical evolution-based scoring function for amino acid substitution cost (BLOSUM62 score). Note that the restriction enforcing two or more Asp and Glu is still present in the both conditions.

In (1), following [2, 3], the amino acid frequency obtained from the genome data is incorporated, as a proxy for the frequency in the proteome, in the cost function as follows:

$$cost(\mathbf{a}) = \sum_c \frac{p(a(c))}{n(a(c))} \sum_{c'} P\left(c'|c\right) d\left(a\left(c\right), a\left(c'\right)\right), \tag{S1}$$

where $p(a)$ is the frequency of amino acid a in the genome data [2] that is normalized so as to $\sum_a p(a) = 61$(i.e., the number of sense codons). $n(c)$ represents the number of synonyms codons in the code $a(c)$. For $p(a) = n(a)$, the original cost in the main text (Eq.1) is obtained. This formulation provides “ soft enforcement ” for the number of synonyms codons for each amino acid: i.e., frequently appearing amino acids in the proteome tend to use more codons.

As shown in S6 Fig, the obtained results with this new cost yield qualitatively the same results as the previous results (Fig 2; main text): The left panel in S6 Fig A shows the scatterplot on the PC1-PC2 space for $cost = 2.9 \pm \Delta$ (the same cost as the SGC). Although, at first glance, there appear to be only three clusters (S6 Fig A left), there exist clear four clusters in the PC1-PC2-PC3 space (S6 Fig A right). Accordingly, the landscape constructed by pilling up the PC1-PC2 scatter plot exhibits only three peaks (S6 Fig B left), but the peak where the SGC belongs to can be divided into two peaks in the PC2-PC3 space (S6 Fig B right). Each cluster (C-F) in S6 Fig A has almost the same configuration (S6 Fig C-F) as the cluster in the original model (Fig 2C-F). The blue cluster C in S6 Fig A has high polar requirement amino acids on the left two columns (S6 Fig C), while the orange cluster D in S6 Fig A tends to possess such amino acids on the fourth column. The green E and red F clusters have high polar requirement amino acids on the upper and lower rows at the third column, respectively (S6 Figs E and F).

In (2), we incorporated an empirical evolution-based scoring function for amino acid substitution (BLOSUM62 score [4]) into the cost function. The new cost function is given as follows:

$$cost(\mathbf{a}) = \sum_c \frac{p(a(c))}{n(a(c))} \sum_{c'} P\left(c'|c\right) d_{BLOSUM}\left(a\left(c\right), a\left(c'\right)\right), \tag{S2}$$

where d_{BLOSUM} is 21×21 symmetric matrix given by BLOSUM62 score [4]. The “soft enforcement” for the number of synonyms codons is also included.

As shown in S7 Fig, the new cost again provides similar results to the original model (Fig 2). Five clusters appear in the PC1-PC2 space for $cost = 3.3 \pm \Delta$ (the same cost as the SGC). The orange cluster in the original model (Fig 2: the cluster D) that codes high polar requirement AAs at the fourth column is split into two clusters D and F in the new model (S7 Fig A; the orange and purple clusters). Although cluster separation between the red (S7 Fig A; cluster (F)) and green cluster (S7 Fig A; cluster (E)) is less clear than the original model, polar requirement configurations in these codes (S7 Figs E and F) are quite similar to the previous results (Figs 2 E and F). Accordingly, the landscape shows five peaks (S7 Fig B).

TABLE I: List of polar-requirement values, from [1]

amino acid	PR
Ala	7.0
Arg	9.1
Asp	13.0
Asn	10.0
Cys	4.8
Glu	12.5
Gln	8.6
Gly	7.9
His	8.4
Ile	4.9
Leu	4.9
Lys	10.1
Met	5.3
Phe	5.0
Pro	6.6
Ser	7.5
Thr	6.6
Trp	5.2
Tyr	5.4
Val	5.6

Reference

[1] Woese CR, Dugre D, Dugre S, Kondo M, Saxinger W. On the fundamental nature and evolution of the genetic code. In: Cold Spring Harbor symposia on quantitative biology. vol. 31. Cold Spring Harbor Laboratory Press; 1966. p. 723–736.
[2] Gilis D, Massar S, Cerf NJ, Rooman M. Optimality of the genetic code with respect to protein stability and amino-acid frequencies. Genome biology. 2001;2(11):1–12.
[3] Novozhilov AS, Wolf YI, Koonin EV. Evolution of the genetic code: partial optimization of a random code for robustness to translation error in a rugged fitness landscape. Biology direct. 2007;2(1):1–24.
[4] Henikoff S, Henikoff JG. Amino acid substitution matrices from protein blocks. Proceedings of the National Academy of Sciences. 1992;89(22):10915–10919.
