# Supplementary material for: Rare-event sampling analysis uncovers the fitness landscape of the genetic code
Source: PLoS Comput Biol. 2023 Apr 17;19(4):e1011034. doi: 10.1371/journal.pcbi.1011034 (PMC10138212; doi:10.1371/journal.pcbi.1011034)
Supplement: S1 Fig — (A) Example of GUG misreading. The star represents a codon to be misread by translational error. Blue, green, and red indicate erroneous changes at the 1st, 2nd, and 3rd bases, respectively. The number in the table indicates the weight for P(c′|c), the probability of misreading c as c′. For example, misreading is twice as likely when the weight is 1 as when it is 0.5. (B) Example of CCA misreading. (PDF) [file pcbi.1011034.s002.pdf]

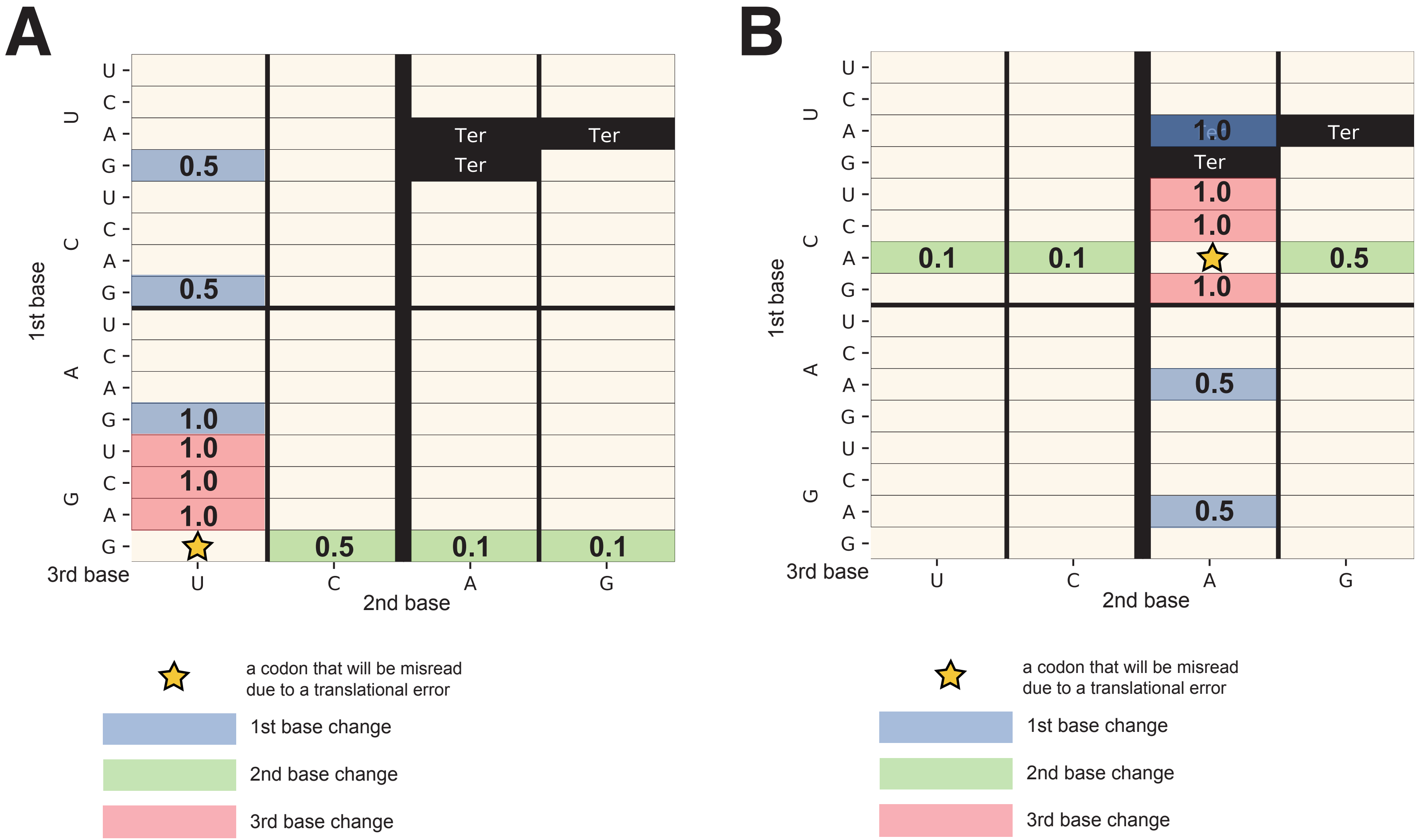

A
B
1st base
2nd base
3rd base
U
C
A
G
Ter
0.5
1.0
0.1
a codon that will be misread
due to a translational error
1st base change
2nd base change
3rd base change
