# Supplementary material for: Rare-event sampling analysis uncovers the fitness landscape of the genetic code
Source: PLoS Comput Biol. 2023 Apr 17;19(4):e1011034. doi: 10.1371/journal.pcbi.1011034 (PMC10138212; doi:10.1371/journal.pcbi.1011034)
Supplement: S2 Fig — (A) Procedure for generating a random code in the ensemble. The 20 different amino acids are randomly assigned numbers from (1) to (20), thus maintaining the SGC block structure that are groups of codons for where the same amino acid is coded. (B) The Standard genetic code (SGC). (C) Example of a random code generated by (A). (PDF) [file pcbi.1011034.s003.pdf]

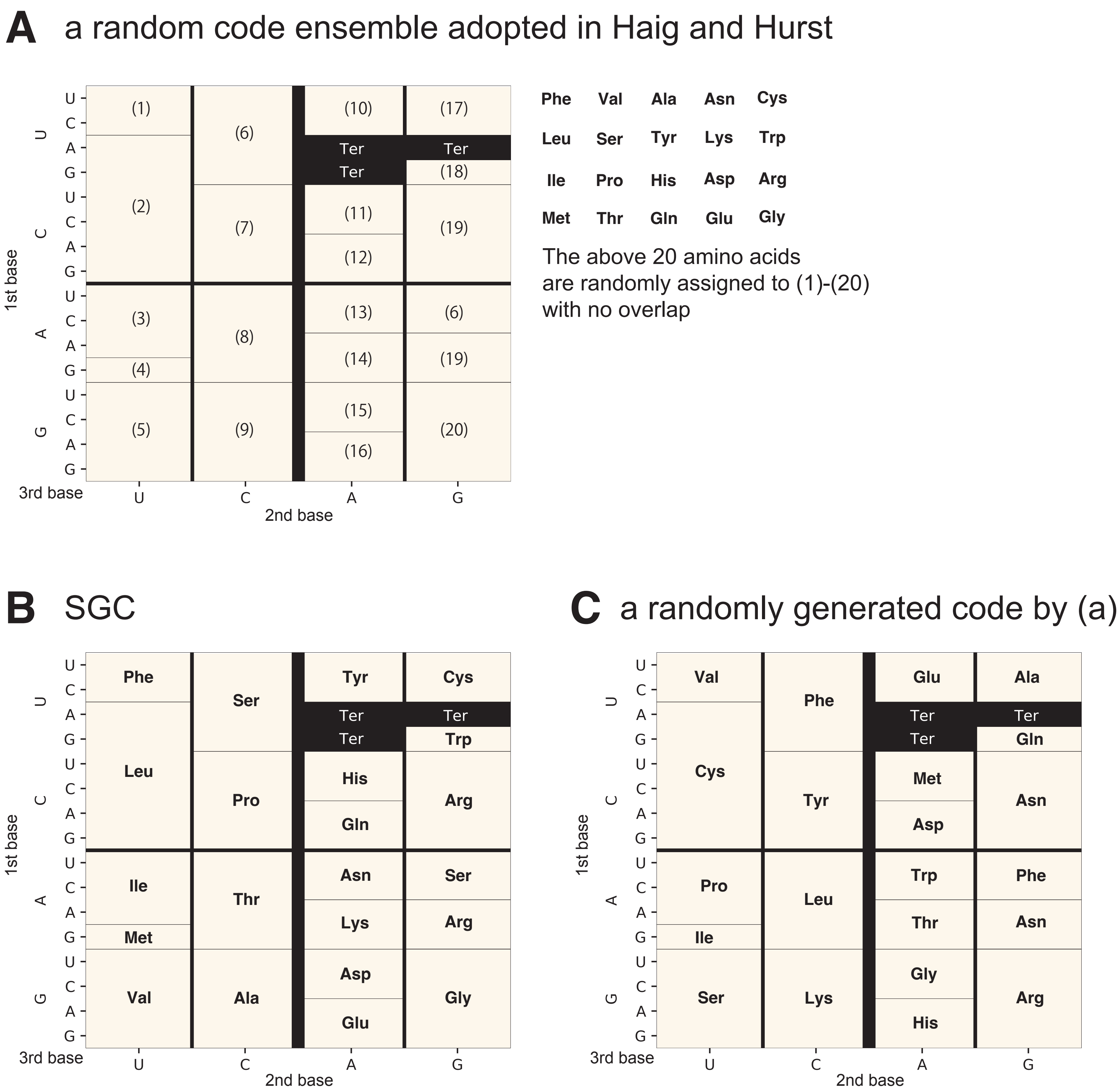
A a random code ensemble adopted in Haig and Hurst
(1) (2) (3) (4) (5) (6) (7) (8) (9) (10) Ter Ter (11) (12) (13) (14) (15) (16) (17) Ter (18) (19) (6) (19) (20)
Phe Val Ala Asn Cys
Leu Ser Tyr Lys Trp
Ile Pro His Asp Arg
Met Thr Gln Glu Gly
The above 20 amino acids
are randomly assigned to (1)-(20)
with no overlap
B SGC
Phe Leu Ile Met Val Ser Pro Thr Ala Tyr Ter Ter His Gln Asn Lys Asp Glu Cys Ter Trp Arg Ser Arg Gly
C a randomly generated code by (a)
Val Cys Pro Ile Ser Phe Tyr Leu Lys Glu Ter Ter Met Asp Trp Thr Gly His Ala Ter Gln Asn Phe Asn Arg
1st base
2nd base
3rd base
U C A G
