# Supplementary figures and images for: Rare-event sampling analysis uncovers the fitness landscape of the genetic code
Source: PLoS Comput Biol. 2023 Apr 17;19(4):e1011034. doi: 10.1371/journal.pcbi.1011034 (PMC10138212; doi:10.1371/journal.pcbi.1011034)

**A**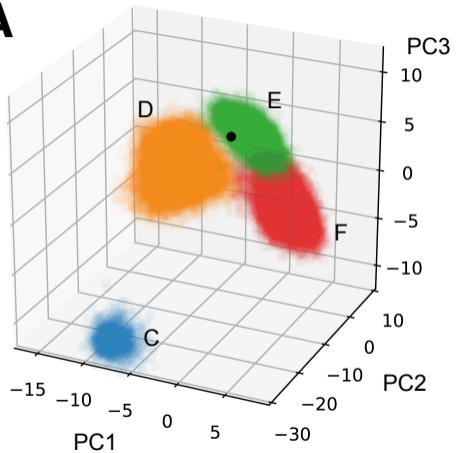**B**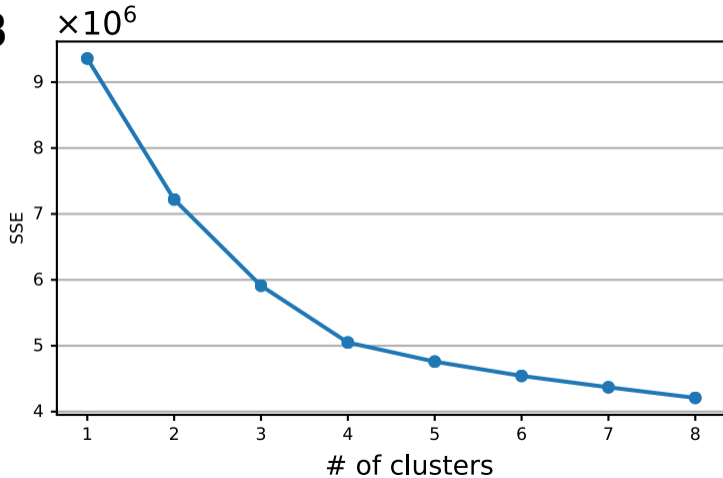

Supplement: S3 Fig — (A) Visualization of the scatter plot Fig 2A in the PC1-PC2-PC3 space. (B) Elbow method for determining the number of the cluster = 4 in Fig 2A. The sum of square error (SSE) is plotted as a function of the number of clusters. The SSE decreases rapidly as the number of clusters increases from 1 to 4, then declines more slowly from 4 to 8 clusters. (PDF) [file pcbi.1011034.s004.pdf]

**A**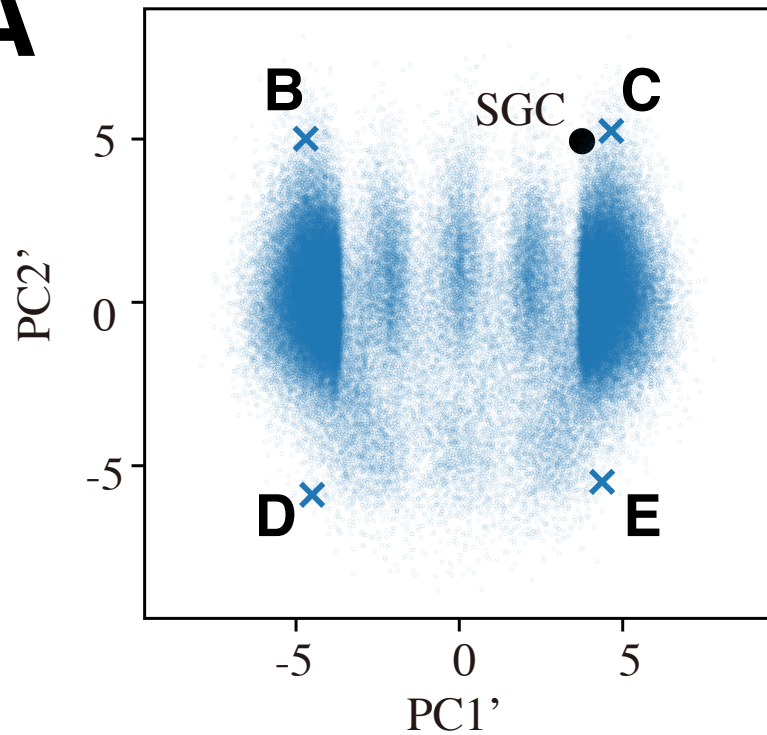**B**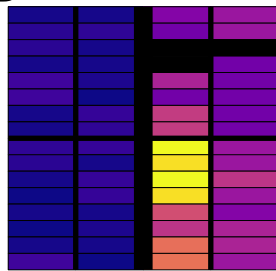**C**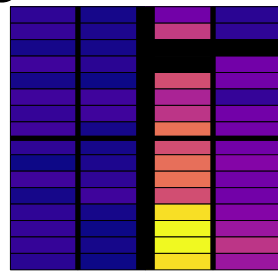**D**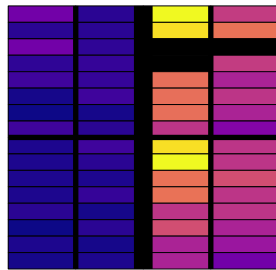**E**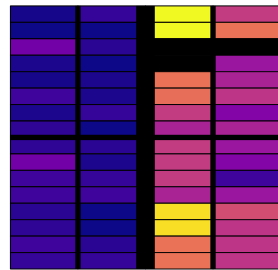

Supplement: S4 Fig — (A) Scatterplot on the PC1-PC2 plane (PC scores: PC1, 31%; PC2, 7%). The symmetrical shape is attributed to the symmetry of the cost function, with respect to the swapping of the bottom four rows and the next four rows. (B-D) Examples of random genetic codes in the four corners in (A). The code in (C) is located near the SGC on the PC1-PC2 plane, and indeed has a similar structure to the SGC. (PDF) [file pcbi.1011034.s005.pdf]

**A**

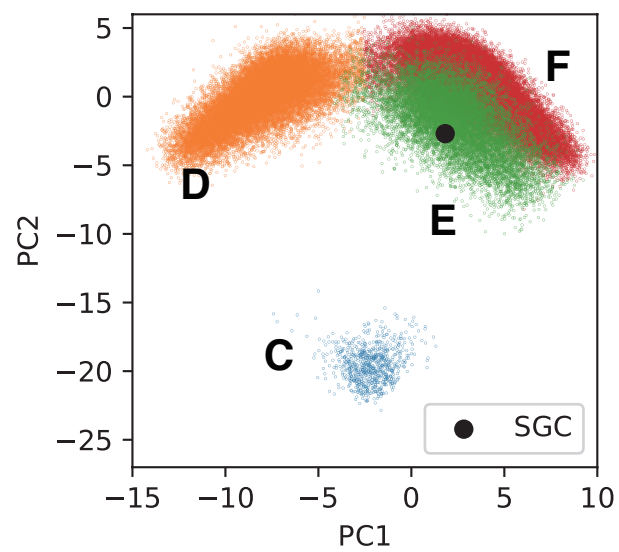

**B**

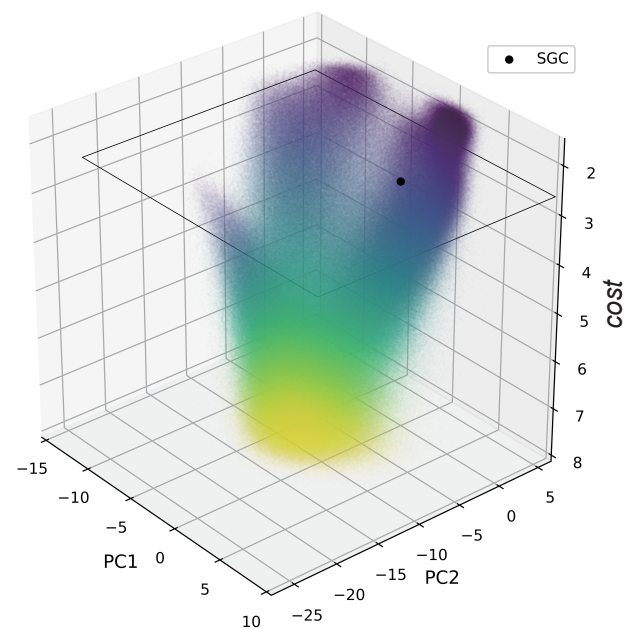

**C**

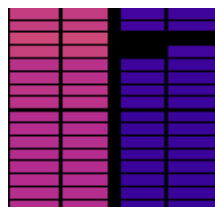

**D**

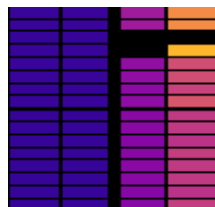

**E**

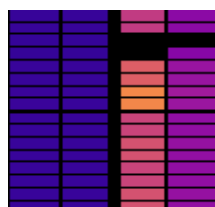

**F**

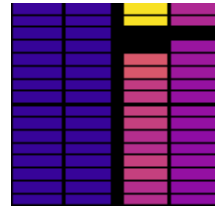

**C'**

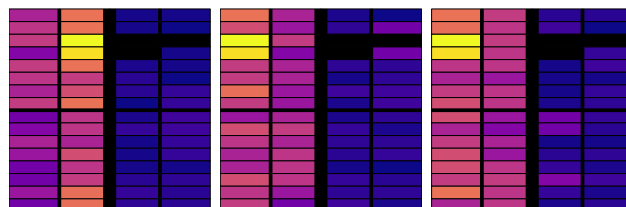

**D'**

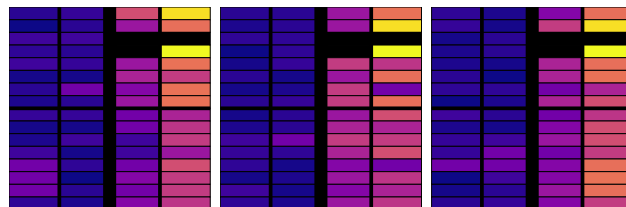

**E'**

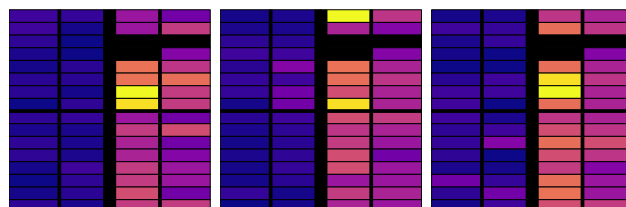

**F'**

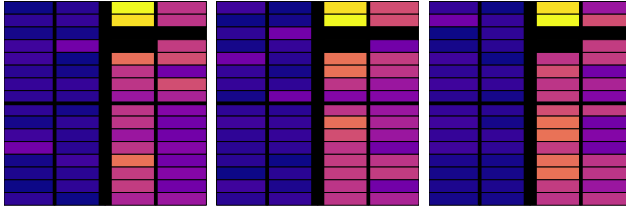

Supplement: S5 Fig — Here, we consider a random genetic code ensemble of genetic codes with 20 different amino acids (i.e., at least one instance of Asp and Glu). (A) Scatterplot of genetic codes with cost values comparable to those of the SGC [2.6 − Δ < cost < 2.6 + Δ (Δ = 0.1)] on the PC1-PC2 plane. (B) Fitness landscape visualized by piling up the scatterplot on the PC1-PC2 plane with different costs on the vertical axis. (C-F) Average structure of the random genetic code in each cluster. (C’-F’) examples of samples belonging to the clusters (C-F) in (A). (PDF) [file pcbi.1011034.s006.pdf]
