# Supplementary material for: Rare-event sampling analysis uncovers the fitness landscape of the genetic code
Source: PLoS Comput Biol. 2023 Apr 17;19(4):e1011034. doi: 10.1371/journal.pcbi.1011034 (PMC10138212; doi:10.1371/journal.pcbi.1011034)
Supplement: S8 Fig — (A) Percentage of genetic codes with one instance of Asp and Glu, against cost. At low costs, almost all of the genetic codes have only one instance of Asp and Glu. (B) Mean and variance of the number of amino acids in the random genetic codes, with cost 2.6 − Δ < cost < 2.6 + Δ (Δ = 0.1), for the same ensemble as in (A). (C) The same plot as in (B), but for the random code ensemble with at least two instances of Asp and Glu in each genetic code. The numbers of amino acids in the SGC are within 2σ of the number obtained using the random ensemble. (PDF) [file pcbi.1011034.s009.pdf]

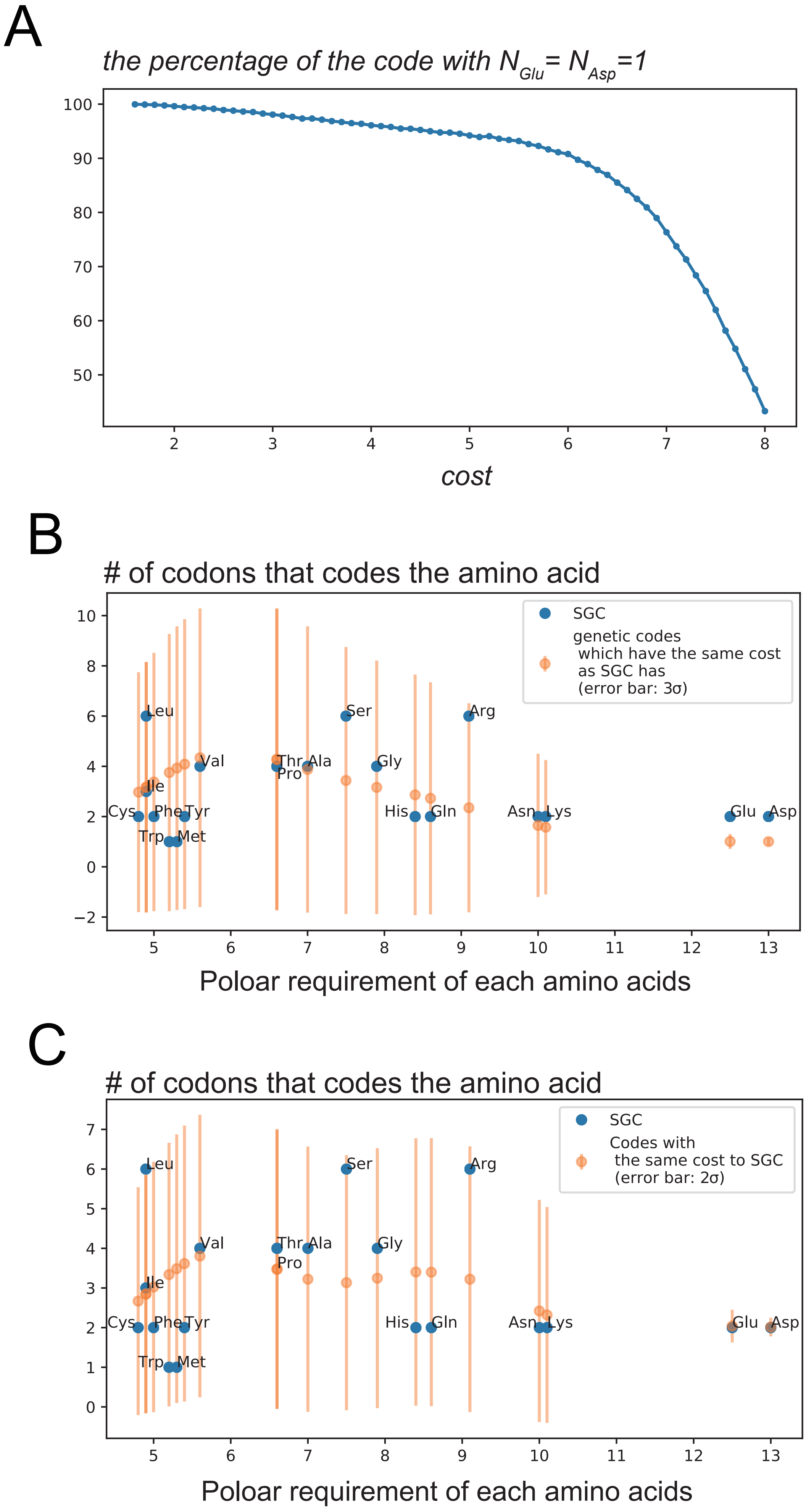
A
the percentage of the code with N_Glu = N_Asp = 1
100
90
80
70
60
50
2
3
4
5
6
7
8
cost
B
# of codons that codes the amino acid
SGC
genetic codes which have the same cost as SGC has (error bar: 3σ)
Leu
Ser
Arg
Val
Thr
Ala
Pro
Gly
Ile
Cys
Phe
Tyr
His
Gln
Asn
Lys
Glu
Asp
Trp
Met
10
8
6
4
2
0
−2
5
6
7
8
9
10
11
12
13
Poloar requirement of each amino acids
C
# of codons that codes the amino acid
SGC
Codes with the same cost to SGC (error bar: 2σ)
Leu
Ser
Arg
Val
Thr
Ala
Pro
Gly
Ile
Cys
Phe
Tyr
His
Gln
Asn
Lys
Glu
Asp
Trp
Met
7
6
5
4
3
2
1
0
5
6
7
8
9
10
11
12
13
Poloar requirement of each amino acids
