# Supplementary material for: Rare-event sampling analysis uncovers the fitness landscape of the genetic code
Source: PLoS Comput Biol. 2023 Apr 17;19(4):e1011034. doi: 10.1371/journal.pcbi.1011034 (PMC10138212; doi:10.1371/journal.pcbi.1011034)
Supplement: Attachment — Submitted filename: point_by_point_reply_20230221b.pdf [file pcbi.1011034.s011.pdf]

Reply to Reviewer #1

We would like to thank the reviewer for the constructive suggestions to improve our manuscript. We have revised the manuscript following the reviewer's suggestions. Our responses to specific comments are shown below.

Comment #1-1: The conclusion that the SGC is more robust than every 10^{20} AGCs is interesting. On the one hand, the discrepancy between this conclusion and the classical "one in a million" claim [1] is due to the fact that Omachi et al. broke the block structure of genetic code when simulating AGCs, which naturally makes AGCs less robust than AGCs generated in previous studies. On the other hand, given the 10^{79} genotype space they explored, there will be ~10^{59} AGCs that are at least as robust as the SGC, which is very surprising.

Response #1-1: We thank the reviewer for the careful evaluation and the encouraging comments. As is written below, we have corrected the manuscript based on the reviewer's suggestions.

Comment #1-2: Does the claim about four major fitness peaks provide us valuable information about the evolutionary origin of the SGC? Probably not yet.
(1) The PCA is performed on a subset of AGCs with similar or lower costs than the SGC. If the PCA is performed on all the genotypes, the clustering pattern could be very different. It is unclear why the authors only use this subset to do the dimensionality reduction.

Response #1-2: The reason we use a subset of random codes (AGCs) for PCA is that the dataset of all possible random codes forms 61 dimensions and therefore cannot be compressed. Selecting low-cost codes allows for meaningful dimension reduction, which enables us to reconstruct the fitness landscape, as shown in Fig. 2b. To clarify this point, we have added the following explanation.

Revision #1-2 (Page 6, Line 195):
Using the sampled data with costs comparable to, or lower than, those of the SGC (i.e., $cost < 2.6 + ¥Delta$, where $¥Delta=¥red{0.1}$), we reduced the 64-dimensional code space to two-dimensional space, via principal component analysis (PCA). This use of a

subset of random codes for PCA is based on our assumption that only low-cost random codes can form a meaningful low-dimensional structure.

Comment #1-3: (2) The first two PCs only explained a relatively small proportion of the variance (~40%). Will including more PCs change the clustering pattern? In other words, there are no justifications and power analysis for just using the first two PCs to explore the connectivity of the peaks in the fitness landscape of genetic codes.

Response #1-3: We thank the reviewer for the valuable comment. We confirmed that no additional patterns were discovered when plotting the scatter plot from Fig. 2a in the PC1-PC2-PC3 space (Fig. S3a), suggesting that the PC1-PC2 plane is sufficient to represent the structure of the low-cost codes. We have added Fig. S3a and revised the manuscript to clarify this feature of low-cost codes.

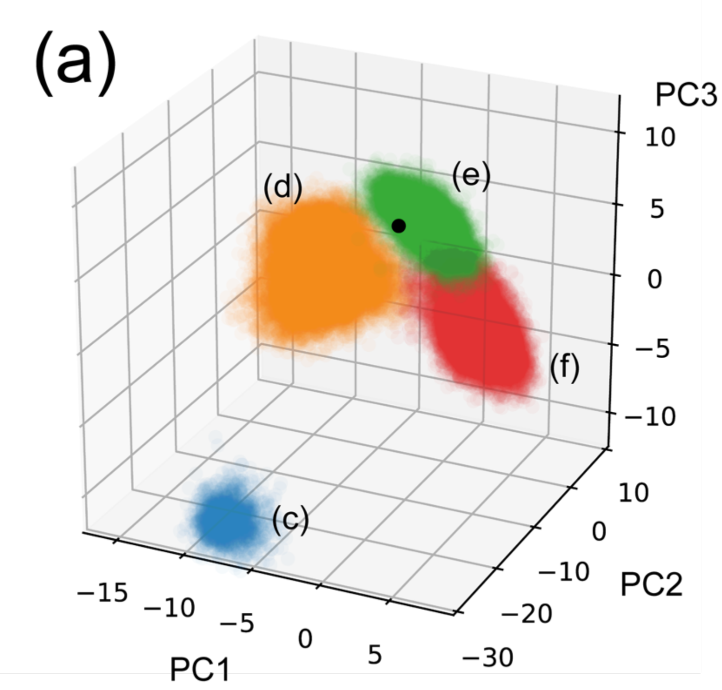

Fig. S3: Cluster number determination for the scatter plot in Fig.2a.

(a) Visualization of the scatter plot Fig. 2a in the PC1-PC2-PC3 space.

Revise #1-3 (Page 6, Line 203):

No further structures were found in the PC1-PC2-PC3 space (Fig. S3a).

Comment #1-4: (3) Given that there are four global peaks existing, why the SGC ends up in this particular peak? Is it just historical contingency or is it because of selection on other aspects of the SGC?

Response #1-4: We thank the reviewer for the constructive criticism. Based on the present analysis, it is difficult to conclude why the SGC evolved into its current form. As the reviewer pointed out, several hypotheses can be proposed: (a) historical contingency may account for its current peak with no underlying reason, (b) the coevolution between code robustness and amino acid synthetic pathways might offer an explanation. For example, a previous study (Higgs, Biology Direct, 2009) proposed a four-column structure of the earliest genetic code, where only Val, Ala, Asp, and Gly were encoded, which could have influenced the evolution towards the present peak. (c) Other selection pressures that were overlooked in the present study could also contribute to its evolution. While the SGC has been discussed to be under selection pressure to minimize changes in polar requirement due to misreading, other factors such as molecular volume, charge, or hydropathy could also impact the code fitness. Integrating these factors might provide an explanation for why the SGC has evolved into its current peak.

While various hypotheses can be proposed, it is important to emphasize that our sampling methodology provide a foundation for exploring these hypotheses. To address these points, we have added the following sentences to the revised manuscript.

Revision #1-3 (Page 10, Line 331):
Likewise, the present analysis provided no answer as to why the SGC stopped at a suboptimal location for a particular peak. Several hypotheses can be made: (a) the SGC has been frozen to the present position during evolution. (b) The code structure at a very early stage of evolution may have influenced the current SGC~¥cite{higgs2009four,wong1975co,koonin2009origin}. (c) We might fail to capture the true code fitness, where the SGC is the global optimal. Selection pressures other than robustness against mistranslations or amino-acid properties other than polar requirements may need to be considered.

Comment #1-5: (4) Since many more genotypes are better than the SGC, why did SGC stop evolution towards a higher peak?

Response #1-5: This point remains open, and similar hypotheses to those in Response #1-4 can be proposed: (a) the SGC may not be optimal and could have been frozen to the present form during evolution, (b) the code structure that originally encoded only a few amino acids in the earliest stages of evolution may still persist in the present SGC, or (c) we may have failed to capture the true code fitness, where the SGC represents the global optimum.

Again, it should be noted that future analysis to explore the above hypotheses can be based on our study and methodology. To mention these points, we revised manuscript as follow.

Revision #1-5 (Page 10, Line 331):
Likewise, the present analysis provided no answer as to why the SGC stopped at a suboptimal location for a particular peak. Several hypotheses can be made: (a) the SGC has been frozen to the present position during evolution. (b) The code structure at a very early stage of evolution may have influenced the current SGC~¥cite{higgs2009four,wong1975co,koonin2009origin}. (c) We might fail to capture the true code fitness, where the SGC is the global optimal. Selection pressures other than robustness against mistranslations or amino-acid properties other than polar requirements may need to be considered.

Comment #1-6: (5) Although the authors mentioned in their discussion that incorporating other parameters, such as codon usage is beyond the scope of this study, the importance and generosity of the findings in the fitness landscape will be significantly impaired without examining it under other conditions. For example, they could use different mistranslation error rates for each codon based on proteomics data, using different amino acid properties to calculate the cost, or considering codon usage [4].

Response #1-6: We thank the reviewer for this constructive suggestion. To demonstrate the generality of our conclusions, we performed additional simulations using two different cost (fitness) functions: (1) a cost function that includes the amino acid frequencies in the genomes of extant organisms, which mimics the frequencies in proteomes, and (2) a cost function that incorporates an empirical, evolution-based scoring function for amino acid substitution cost (BLOSUM62 score). Note that the restriction of having two or more Asp and Glu is still present in both conditions.

In (1), following [4,8], the amino acid frequency obtained from the genome data

is incorporated, as a proxy for the frequency in the proteome, in the cost function as follows:

$$\mathrm{cost}(\mathbf{a}) = \sum_{\mathrm{c}} \frac{\mathrm{p}(\mathrm{a}(\mathrm{c}))}{n(a(\mathrm{c}))} \sum_{\mathrm{c}'} \mathrm{P}(\mathrm{c}'|\mathrm{c}) \mathrm{d}\big(\mathrm{a}(\mathrm{c}), \mathrm{a}(\mathrm{c}')\big),$$

where $\mathrm{p}(\mathrm{a})$ is the frequency of amino acid a in the genome that is normalized so that $\sum_{\mathrm{a}} \mathrm{p}(\mathrm{a}) = 61$ (i.e., the number of sense codons). $\mathrm{n}(\mathrm{c})$ represents the number of synonyms codons in the code $\mathrm{a}(\mathrm{c})$. For $\mathrm{p}(\mathrm{a}) = \mathrm{n}(\mathrm{a})$, the original cost in the main text (Eq.1) is obtained. This formulation provides a “soft enforcement” for the number of synonymous codons for each amino acid. In other words, frequently appearing amino acids in the proteome tend to use more codons.

As shown in Fig.S6, the obtained results with this cost function yield qualitatively the same results as the previous results in Fig.2. The left panel in Fig.S6a shows the scatterplot on the PC1-PC2 space for cost = 2.9 $\pm\Delta$ (the same cost as the SGC). Although, at first glance, there appear to be only three clusters (Fig.S6a left), there exist clear four clusters in the PC1-PC2-PC3 space (Fig.S6a right). Accordingly, the landscape constructed by pilling up the PC1-PC2 scatter plot exhibits only three peaks (Fig.S6b left), but the peak where the SGC belongs to can be further divided into two peaks in the PC2-PC3 space (Fig.S6b right). Each cluster (c)-(f) in Fig.S6a has almost same configuration (Fig.S6c-f) to the cluster in the original model (Fig.2c-f). The blue cluster (c) in Fig.S6a has high polar requirement amino acids on the left two columns (Fig.S6c), while the orange cluster (d) in Fig.S6a tends to possess such amino acids on the fourth column. The green (e) and red (f) clusters have high polar requirement AAs on the upper and lower rows at third column, respectively (Figs.S6e and f).

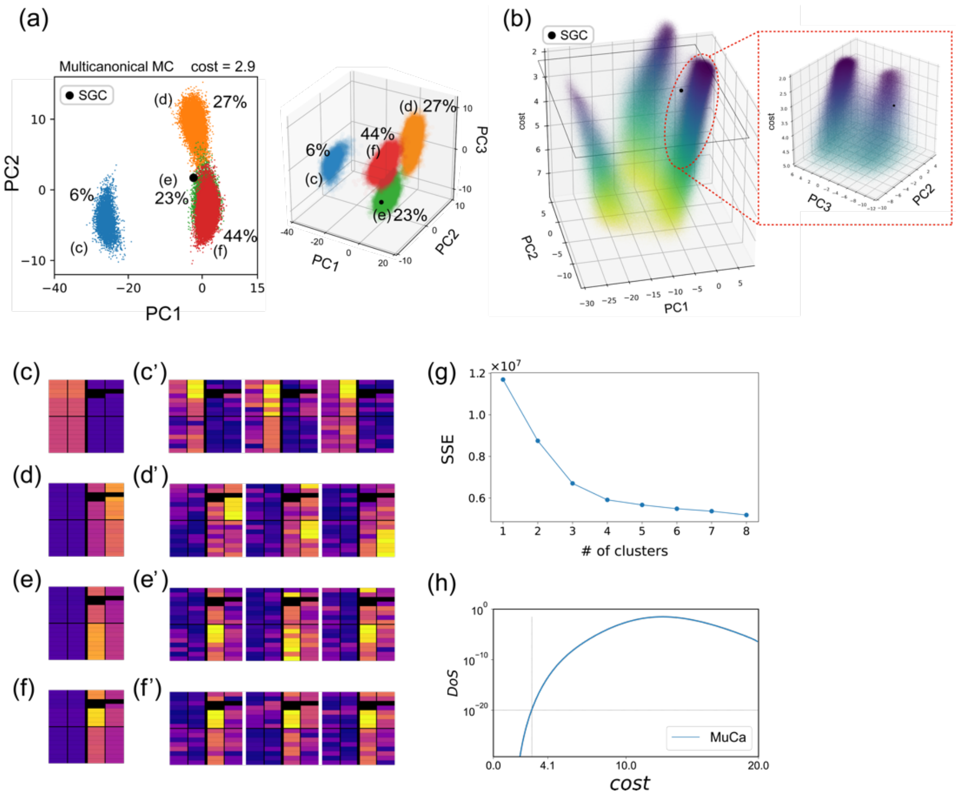

Fig. S6 Fitness landscape under a random genetic code ensemble incorporating the amino acid frequency in the genomes.} The cost function defined by Eq.~S1 in the supplemental text is used. (a) Genetic codes with comparable cost values to the SGC, where $2.9-¥Delta<cost <2.9+¥Delta$ ($¥Delta=0.1$) in the PC1-PC2 space (left panel) and in the PC1-PC2-PC3 space (the contribution ratios: PC1, 31¥%, PC2, 18¥%, PC3 9¥%) (right panel). Principal component analysis and k-means clustering were performed using the sampled data with a cost value of $cost <2.9+¥Delta$. (b) Fitness landscape visualized by piling up the scatterplot on the PC1-PC2 plane, with costs decreasing on the vertical axis (left panel). The peak that the SGC belongs to in the PC1-PC2 plane is also shown in the PC2-PC3 plane (right panel). (c-f) Average structure of the genetic code in each cluster. (c'-f') examples of samples belonging to the clusters (c-f) in (a). (g) Elbow method for determining the number of clusters = 4 in (a). The sum of square error (SSE) is plotted as a function of the number of clusters. (h) Cost density-of-states function for the ensemble estimated by the multicanonical Monte Carlo.

In (2), we incorporated an empirical evolution-based scoring function for amino acid substitution (BLOSUM62 score [31]) into the cost function. The new cost function is given as follows:

$$\mathrm{cost}(\mathbf{a}) = \sum_{c} \frac{p(a(c))}{n(a(c))} \sum_{c'} P(c'|c) d_{\mathrm{BLOSUM}}\big(a(c), a(c')\big),$$

where d_{BLOSUM} is 21 x 21 symmetric matrix given by BLOSUM62 score [31]. A "soft enforcement" for the number of synonyms codons is also included.

As shown in Fig.S7, the new cost again provides similar results to the original model (Fig.2). Five clusters appear in the PC1-PC2 space for cost = 3.3 ±Δ (the same cost as the SGC). The orange cluster in the original model (Fig.2: the cluster (d)) that encode high polar requirement AAs at fourth column is split into two clusters (d) and (f) in the new model (Fig.S7a; the orange and purple clusters). Although cluster separation between the red (Fig.S7a; cluster (f)) and green cluster (Fig.S7a; cluster (e)) is less clear than the original model, polar requirement configurations in these codes (Figs.S7 e and f) are quite similar to the previous results (Figs.2 e and f). Accordingly, the landscape shows five peaks (Fig.S7b).

The analysis using conditions (1) and (2) suggests that our findings about the fitness landscape are robust with respect to the details of the cost function. To further discuss the generality of the results, we have added Fig. S6, Fig. S7, and supplemental text to the revised manuscript, and have made the necessary revisions as follows.

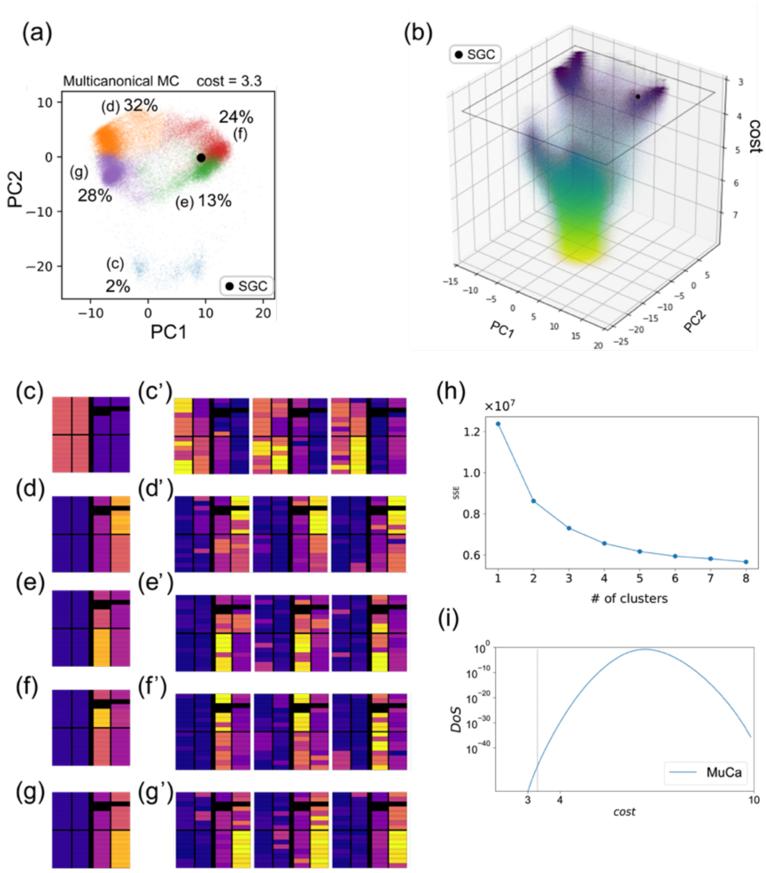

Fig. S7 Fitness landscape under a random genetic code ensemble incorporating BLOSUM62 score. The cost function defined by Eq.~S2 in the supplemental text is used. (a) Genetic codes with comparable cost values to the SGC, where $3.3-¥Delta<cost <3.3+¥Delta$ ($¥Delta=0.1$) in the PC1-PC2 space (the contribution ratios: PC1, 21¥%, PC2, 8¥%). Principal component analysis and k-means clustering were performed using the sampled data with a cost value of $cost <3.3+¥Delta$. (b) Fitness landscape visualized by piling up the scatterplot on the PC1-PC2 plane, with costs decreasing on the vertical axis. (c-g) Average structure of the genetic code in each cluster. (c'-g') examples of samples belonging to the clusters (c-g) in (a). (h) The sum of square error (SSE) in the elbow method is plotted as a function of the number of clusters. (i) Cost density-of-states function for the ensemble estimated by the multicanonical Monte Carlo.

Revision #1-6 (Page 7, Line 219):

So far, we considered an ensemble containing at least two Asp and two Glu (i.e., condition (4) in the Methods section). By performing additional simulations, we also confirmed that our results do not depend on this condition. A closely similar fitness landscape with four peaks appears even in the absence of the condition (see Supplemental text and Fig.S5). Further, we performed simulations with two different cost (fitness) functions: (1) the cost including amino acid frequencies in the genome in extant organisms, and (2) the cost incorporating an empirical evolution-based scoring function for amino acid substitution (BLOSUM62 score~¥cite{henikoff1992amino}). In both cases, similar fitness landscapes with multi-peaks were confirmed. In cost incorporating BLOSUM score, the orange cluster in the original model (Fig.2: the cluster (d)) that codes high polar requirement amino acids at the fourth column is split into two clusters (d) and (f) under this cost function (Fig.S7a; the orange and purple clusters). However, the amino acid configuration for each peak is similar to the original model (Fig.2c-f).

(Page 10, Line 314):

We also examined an ensemble without the condition that two Alu or Glu must be included (Fig.S5).

Further, the additional simulations were performed with two different cost functions: a cost incorporating amino acid frequencies in genome data (Fig.S6) and a cost based on BLOSUM62 score~¥cite{henikoff1992amino} (Fig.S7). In each case, similar fitness landscapes with multiple peaks were identified (Figs.S5-S7; see also supplemental text). From these results, we concluded the presence of four (or five) major peaks in the fitness landscape of the genetic code.

Reference:
[4] Gilis, et al. (2001) Genome biology, 2, 1-12.
[8] Novozhilov, et al. Biology direct. 2007;2(1):1–24.
[31] Henikoff & Henikoff (1992). Proceedings of the National Academy of Sciences, 89(22), 10915-10919.

Comment #1-7: The third point they made is interesting and general to any kind of fitness landscape. This point is essentially talking about the overall shape of peaks, some peaks could have a large basin but become much narrower when reaching the top.

Overall, I think the first and third points are clear. However, there are unclear issues about point 2 and other questions that should be explained and justified before acceptance for publication."

Response #1-7: We would like to express our gratitude to the reviewer for carefully and positively evaluating our work. We believe that the previously unclear issues pointed out by the reviewer have now been resolved, and that our main claim is now justified.

Comment #1-8: Other questions:

(1) For Fig. 2a, why only AGCs with a cost similar to the SGC are plotted while the PCA and clustering is performed using sample data with a cost similar or lower than the SGC?

(2) There is a sentence repetition in line 240.

Response #1-8:

(1) Since we focused on the structure of the genetic code in the low-cost region, we performed PCA and clustering using only data with a cost below that of the SGC. Furthermore, using all data below the cost of the SGC can increase the number of samples and result in more robust clustering compared to using only data with a similar cost to the SGC. However, in Fig. 2a, which corresponds to a cross-section in Fig. 2b, we used only data with a similar cost to the SGC to facilitate an understanding of the structure of the clusters. We adopted the present visualization in Fig. 2a for these reasons.

(2) We thank the reviewer for pointing this out. We have corrected it.

Reply to Reviewer #2

We would like to thank the reviewer for the constructive suggestions to improve our manuscript. We have revised the manuscript following these suggestions. Our responses to specific comments are shown below.

Comment #2-1: Omachi, Saito & Chikara Furusawa offer an estimate for the frequency of low-cost genetic codes using an advanced technique, multicanonical Monte Carlo sampling. They estimate that random genetic codes as robust as the Standard Genetic Code (SGC) with respect to hydrophobicity requirement of misread amino acids have frequency on the order of 10^-20; much lower than the previous estimates by Freeland & Hurst (10^-6).

Omachi et al. analysis is very interesting and certainly deserves to be published. There are, however, several issues that need to be addressed in the revision; at least at the level of the text editing, but, possibly, also by modification of the key expression in eq (1).

Response #2-1: We would like to thank the reviewer for careful and positive evaluation of our study. We believe that the previous unclear points raised by the reviewer have now been resolved and that our main claims have been justified.

Comment #2-2: The core question here is what is a "random genetic code" (rGC)? The authors criticize Freeland & Hurst for retaining the block structure of SGC in their rGCs, noting that this is severely limiting the number of rGC choices. Instead they adopt a different way to generate block-like structure of robust rGCs, using the misreading probability (eq 2) that mimics the way SGC codons function in extant ribosomes. This "soft enforcement" is, arguably, a much better choice, but it should be clearly understood and explicitly stated that this is just a different choice of what SGC features are fundamental and which are dispensable.

Response #2-2: We would like to express our gratitude to the reviewer for the suggestion to improve the logical flow of our manuscript. Following this advice, we have substantially rewritten the Methods section so that the conditions that we hypothesize a genetic code must satisfy are presented first (please see line 111 at page 5).

Comment #2-3: Notably, while the authors initially introduce a very general definition of rGC (ll. 35-40), later they add some additional limitations (ll. 141-151) on the ground of being "compatible with our goal of visualizing the fitness landscape to which the SGC belongs". Conceptually, this is the same as enforcing the block structure, just a different choice of what SGC features are judged fundamental and what are deemed dispensable. Incidentally, I find that forcing the >=2 codon series for Asp and Glu is an unreasonable restriction in the context of this work; this restriction does not naturally arise from the hydrophobicity match requirements and if such rGCs are "better" than SGC, so be it.

Response #2-3: We apologize that we accidentally deleted a part of the text that our results do not change without this condition; the supplemental text and Fig.S5 demonstrated the simulation results without the condition about Asp and Glu. We confirmed that qualitatively similar results including appearance of the four fitness peaks are obtained (Fig.S5) even without the restriction in the codon number of Asp and Glu. To explain this point, we added the explanation at L.219 and the supplemental text in the revised manuscript.

As pointed out by the reviewer, the restriction of having two Asp and Glu does not naturally arise from arguments of code robustness and is rather artificial. We hypothesized that this condition would be present as a separate selection pressure from the robustness of the code. This is because, without these conditions, the structure of a random code with a cost comparable to the SGC would be dissimilar to the current configuration of the SGC. We have thoroughly revised the supplemental text to clarify the above points.

Revision #2-3 (Page 7, Line 219):
So far, we considered an ensemble containing at least two Asp and two Glu (i.e., condition (4) in the Methods section). By performing additional simulations, we also confirmed that our results do not depend on this condition. A closely similar fitness landscape with four peaks appears even in the absence of the condition (see Supplemental text and Fig.S5).

Comment #2-4: I suggest to start the Methods section with a concise description of what the authors consider the fundamental properties of SGC that they require to be present in their rGCs (encoding the the full repertoire of AAs, with or without limitations on some of them, the specific form of misreading probability function, other limitations on

terminators etc) and explain how their choices are different from those of their predecessors.

Response #2-4: Following the reviewer's suggestion, we have substantially rewritten the Methods section so that the conditions that we hypothesize a genetic code must satisfy are presented first (please see L.111).

Comment #2-5: Besides the purely presentation level improvements, there are two ways that, I believe, could considerably improve the current analysis. First is going beyond the hydrophobicity requirement (that leads to solo-[DE] rGCs outperforming the SGC) and incorporating an empirical evolution-based scoring function for amino acid substitution cost (the easiest choice would be to incorporate alignment-derived log-odds matrices such as BLOSUM62 or PAM70). Second, assuming amino acid frequencies, mimicking those in the proteomes of extant organisms, would provide "soft enforcement" for the distribution of the size of the codon series (frequent AAs tend to use more codons), and also provide a way to meaningfully include the termination misreading cost that is ignored now (note how the SGC terminator codons are encoded in the same block with rarest amino acids). I realize that this would require a complete reanalysis, so I consider these requests to be optional (but highly recommended).

Response #2-5: We thank the reviewer for this constructive suggestion. To demonstrate the generality of our conclusions, we performed additional simulations using two different cost (fitness) functions: (1) a cost function that includes the amino acid frequencies in the genomes of extant organisms, which mimics the frequencies in proteomes, and (2) a cost function that incorporates an empirical, evolution-based scoring function for amino acid substitution cost (BLOSUM62 score). Note that the restriction of having two or more Asp and Glu is still present in both conditions.

In (1), following [4,8], the amino acid frequency obtained from the genome data is incorporated, as a proxy for the frequency in the proteome, in the cost function as follows:

$$\mathrm{cost}(\mathbf{a}) = \sum_{c} \frac{\mathrm{p}(\mathrm{a}(\mathrm{c}))}{\mathrm{n}(\mathrm{a}(\mathrm{c}))} \sum_{c'} \mathrm{P}(\mathrm{c'}|\mathrm{c})\mathrm{d}\big(\mathrm{a}(\mathrm{c}), \mathrm{a}(\mathrm{c'})\big),$$

where $\mathrm{p}(\mathrm{a})$ is the frequency of amino acid a in the genome that is normalized so that

$\sum_a p(a) = 61$ (i.e., the number of sense codons). $n(c)$ represents the number of synonyms codons in the code $a(c)$. For $p(a) = n(a)$, the original cost in the main text (Eq.1) is obtained. This formulation provides a “soft enforcement” for the number of synonymous codons for each amino acid. In other words, frequently appearing amino acids in the proteome tend to use more codons.

As shown in Fig.S6, the obtained results with this cost function yield qualitatively the same results as the previous results in Fig.2. The left panel in Fig.S6a shows the scatterplot on the PC1-PC2 space for cost = 2.9 ±Δ (the same cost as the SGC). Although, at first glance, there appear to be only three clusters (Fig.S6a left), there exist clear four clusters in the PC1-PC2-PC3 space (Fig.S6a right). Accordingly, the landscape constructed by pilling up the PC1-PC2 scatter plot exhibits only three peaks (Fig.S6b left), but the peak where the SGC belongs to can be further divided into two peaks in the PC2-PC3 space (Fig.S6b right). Each cluster (c)-(f) in Fig.S6a has almost same configuration (Fig.S6c-f) to the cluster in the original model (Fig.2c-f). The blue cluster (c) in Fig.S6a has high polar requirement amino acids on the left two columns (Fig.S6c), while the orange cluster (d) in Fig.S6a tends to possess such amino acids on the fourth column. The green (e) and red (f) clusters have high polar requirement AAs on the upper and lower rows at third column, respectively (Figs.S6e and f).

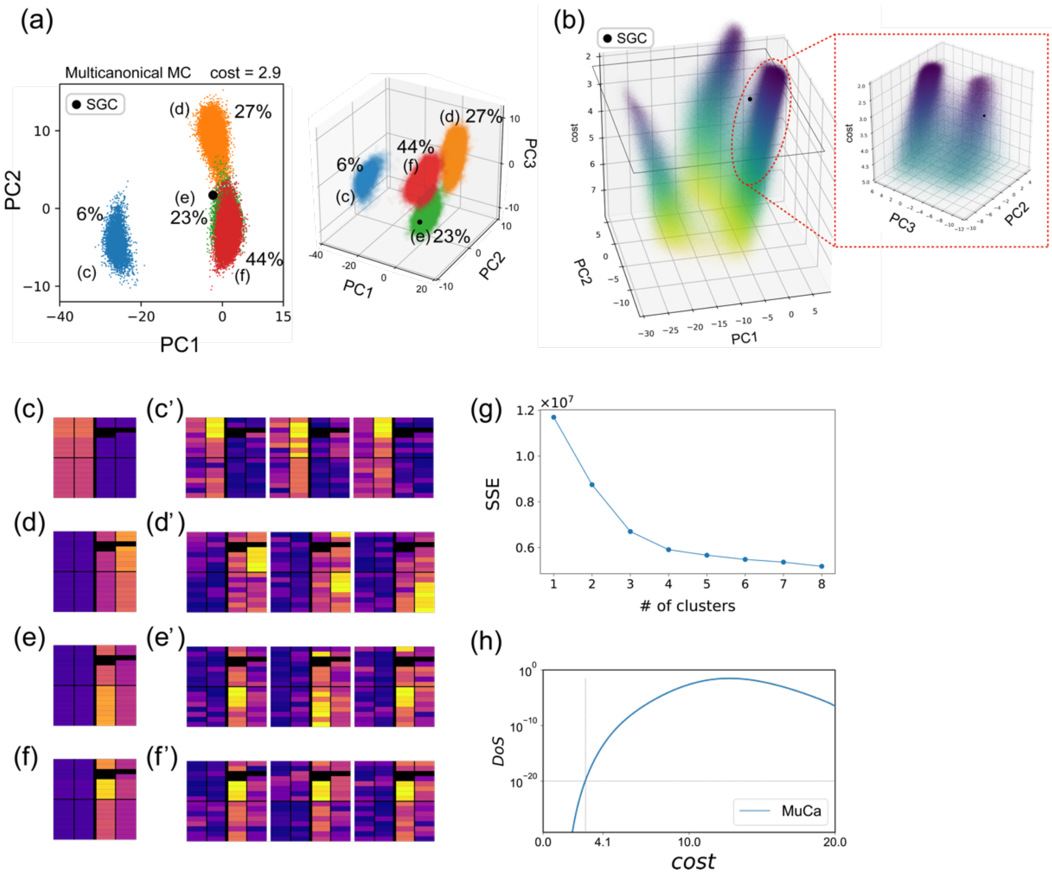

Fig. S6 Fitness landscape under a random genetic code ensemble incorporating the amino acid frequency in the genomes.} The cost function defined by Eq.~S1 in the supplemental text is used. (a) Genetic codes with comparable cost values to the SGC, where $2.9-¥Delta<cost <2.9+¥Delta$ ($¥Delta=0.1$) in the PC1-PC2 space (left panel) and in the PC1-PC2-PC3 space (the contribution ratios: PC1, 31¥%, PC2, 18¥%, PC3 9¥%) (right panel). Principal component analysis and k-means clustering were performed using the sampled data with a cost value of $cost <2.9+¥Delta$. (b) Fitness landscape visualized by piling up the scatterplot on the PC1-PC2 plane, with costs decreasing on the vertical axis (left panel). The peak that the SGC belongs to in the PC1-PC2 plane is also shown in the PC2-PC3 plane (right panel). (c-f) Average structure of the genetic code in each cluster. (c'-f') examples of samples belonging to the clusters (c-f) in (a). (g) Elbow method for determining the number of clusters = 4 in (a). The sum of square error (SSE) is plotted as a function of the number of clusters. (h) Cost density-of-states function for the ensemble estimated by the multicanonical Monte Carlo.

In (2), we incorporated an empirical evolution-based scoring function for amino acid substitution (BLOSUM62 score [31]) into the cost function. The new cost function is given as follows:

$$\mathrm{cost}(\mathbf{a}) = \sum_{\mathrm{c}} \frac{\mathrm{p}(\mathrm{a}(\mathrm{c}))}{\mathrm{n}(\mathrm{a}(\mathrm{c}))} \sum_{\mathrm{c}'} \mathrm{P}(\mathrm{c}'|\mathrm{c}) \mathrm{d}_{\mathrm{BLOSUM}}\big(\mathrm{a}(\mathrm{c}), \mathrm{a}(\mathrm{c}')\big),$$

where $\mathrm{d}_{\mathrm{BLOSUM}}$ is 21 x 21 symmetric matrix given by BLOSUM62 score [31]. A "soft enforcement" for the number of synonyms codons is also included.

As shown in Fig.S7, the new cost again provides similar results to the original model (Fig.2). Five clusters appear in the PC1-PC2 space for cost = 3.3 $\pm\Delta$ (the same cost as the SGC). The orange cluster in the original model (Fig.2: the cluster (d)) that encode high polar requirement AAs at fourth column is split into two clusters (d) and (f) in the new model (Fig.S7a; the orange and purple clusters). Although cluster separation between the red (Fig.S7a; cluster (f)) and green cluster (Fig.S7a; cluster (e)) is less clear than the original model, polar requirement configurations in these codes (Figs.S7 e and f) are quite similar to the previous results (Figs.2 e and f). Accordingly, the landscape shows five peaks (Fig.S7b).

The analysis using conditions (1) and (2) suggests that our findings about the fitness landscape are robust with respect to the details of the cost function. To further discuss the generality of the results, we have added Fig. S6, Fig. S7, and supplemental

text to the revised manuscript, and have made the necessary revisions as follows.

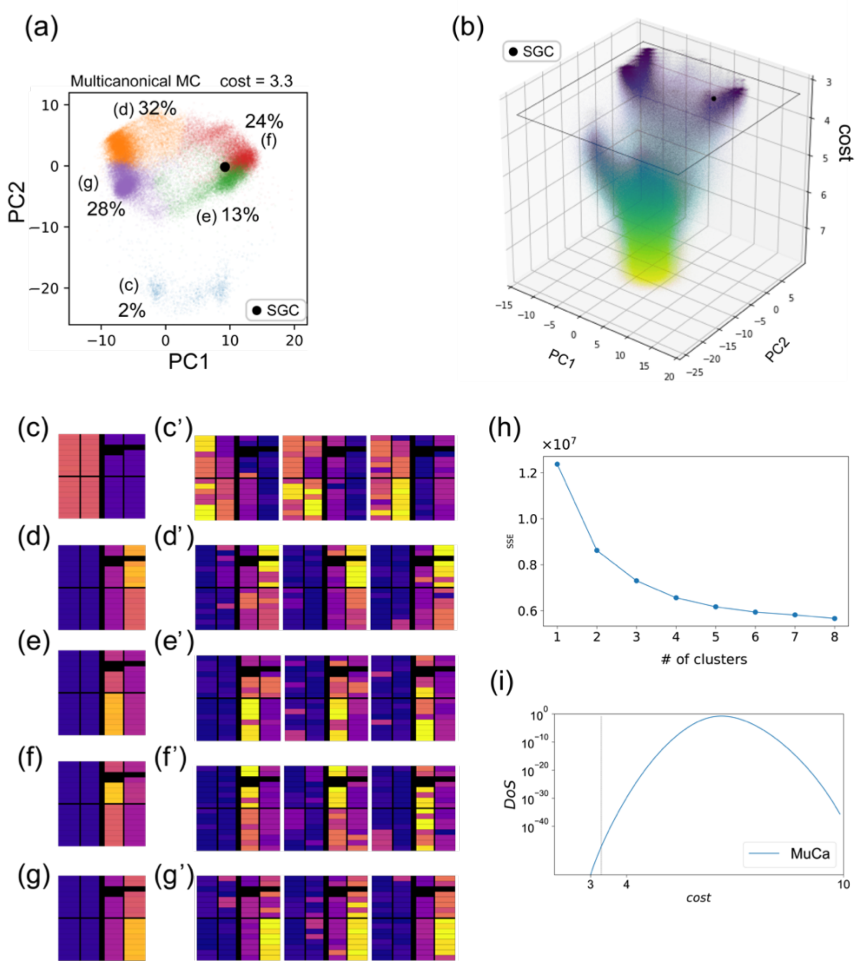

Fig. S7 Fitness landscape under a random genetic code ensemble incorporating BLOSUM62 score. The cost function defined by Eq.~S2 in the supplemental text is used. (a) Genetic codes with comparable cost values to the SGC, where $3.3-¥Delta<cost <3.3+¥Delta$ ($¥Delta=0.1$) in the PC1-PC2 space (the contribution ratios: PC1, 21¥%, PC2, 8¥%). Principal component analysis and k-means clustering were performed using the sampled data with a cost value of $cost <3.3+¥Delta$. (b) Fitness landscape visualized by piling up the scatterplot on the PC1-PC2 plane, with costs decreasing on the vertical axis. (c-g) Average structure of the genetic code in each cluster. (c'-g') examples of samples belonging to the clusters (c-g) in (a). (h) The sum of square error (SSE) in the elbow method is plotted as a function of the number of clusters. (i) Cost density-of-states function for the ensemble estimated by the multicanonical Monte Carlo.

Revision #2-5 (Page 7, Line 223):

Further, we performed simulations with two different cost (fitness) functions: (1) the cost

including amino acid frequencies in the genome in extant organisms, and (2) the cost incorporating an empirical evolution-based scoring function for amino acid substitution (BLOSUM62 score~¥cite{henikoff1992amino}). In both cases, similar fitness landscapes with multi-peaks were confirmed. In cost incorporating BLOSUM score, the orange cluster in the original model (Fig.2: the cluster (d)) that codes high polar requirement amino acids at the fourth column is split into two clusters (d) and (f) under this cost function (Fig.S7a; the orange and purple clusters). However, the amino acid configuration for each peak is similar to the original model (Fig.2c-f).

(Page 10, Line 314):

We also examined an ensemble without the condition that two Alu or Glu must be included (Fig.S5). Further, the additional simulations were performed with two different cost functions: a cost incorporating amino acid frequencies in genome data (Fig.S6) and a cost based on BLOSUM62 score~¥cite{henikoff1992amino} (Fig.S7). In each case, similar fitness landscapes with multiple peaks were identified (Figs.S5-S7; see also supplemental text). From these results, we concluded the presence of four (or five) major peaks in the fitness landscape of the genetic code.

Reference:

[4] Gilis, et al. (2001) Genome biology, 2, 1-12.

[8] Novozhilov, et al. Biology direct. 2007;2(1):1–24.

[31] Henikoff & Henikoff (1992). Proceedings of the National Academy of Sciences, 89(22), 10915-10919.

Reply to Reviewer #3

We would like to thank the reviewer for the constructive suggestions to improve our manuscript. We have revised the manuscript following these suggestions. Our responses to specific comments are shown below.

Comment #3-1: Although the application of this method is an advance in this field, the subject and the biological problem is not new because previous studied already conducted the random selection of codes but in much smaller number. The applied method is also not new because it was previously developed in statistical physics, protein folding and gene regulatory network evolution. Nevertheless, it was used to other problem. Although the study significantly increased the searched space of random codes, it is still a very small fraction in comparison to all possible codes, i.e. 10^83 or 10^59 depending on the assumption on the code structure.

Response #3-1: We would like to thank the reviewer for the careful attention to our work and constructive criticisms. As the reviewer pointed out, both the biological problem and the method used in this study are not new. However, history of science has shown that unprecedented combinations of problems and methods have often resulted in innovative discoveries. In this study, we introduced the multicanonical Monte Carlo method for the first time to investigate genetic code evolution, leading to a considerable expansion of the search space for random codes. While the search space remains smaller than the total possible space, our analysis revealed the configurations of low-cost codes and the fitness landscape of the genetic code, which was unachievable using conventional sampling methods. These findings have paved the way for a more profound understanding of genetic code evolution.

Comment #3-2: The paper can be interesting, however, the authors ignored in Introduction studies that performed optimization of the SGC using various genetic and evolutionary algorithms. The studies should be cited in the background of current studies of the genetic code:

- ✓ Błażej, P., Wnetrzak, M., Mackiewicz, P., 2016. The role of crossover operator in evolutionary-based approach to the problem of genetic code optimization. Biosystems 150, 61-72.

- ✓ Błażej, P., Wnetrzak, M., Mackiewicz, D., Mackiewicz, P., 2018. Optimization of the standard genetic code according to three codon positions using an evolutionary algorithm. PLoS One, 13(8), e0201715.
- ✓ Santos, J., Monteagudo, A., 2011. Simulated evolution applied to study the genetic code optimality using a model of codon reassignments. BMC Bioinformatics, 12, 56.
- ✓ Wnetrzak, M., Błażej, P., Mackiewicz, D., Mackiewicz, P., 2018. The optimality of the standard genetic code assessed by an eight-objective evolutionary algorithm. BMC Evolutionary Biology 18, 192.

Response #3-2: We thank the reviewers for bringing these crucial references to our attention. We agree that these references should be referred to in the paper and have accordingly incorporated them into the revised manuscript.

Revision #3-2 (Page 2, Line 20):
Two different approaches have been used to analyze the fitness landscape of the genetic code: The first is a random sampling-based approach, which has shown how unlikely a code with a high fitness comparable to SGC appears by chance[2-6,8,9], or the existence of a huge number of local fitness peaks[8], etc. The second is an optimization-based approach. For example, studies using evolutionary algorithms have also demonstrated that the SGC is not at the global optimum[10-13]

Comment #3-3: The studies showed that the SGC is not perfectly optimized as it could be expected from the adaptive hypothesis. It would be good to compare the statistical approach (random sampling of codes) with the engineering approach (finding optimal codes in genetic and evolutionary algorithms) in the analysis of adaptation of the SGC.

Response #3-3: We completely agree with the reviewer that comparing the engineering approach (i.e., optimization approach) and random sampling approach is important for comprehending genetic code evolution. The optimization approach entails constructing genetic codes that minimize a given cost function. It enables us to discuss the "optimal strategy" for encoding amino acids and the extent to which the optimal code deviates from the standard genetic code (SGC). This approach is appropriate when investigating optimal codes and their evolutionary trajectories. On the other hand, the multicanonical Monte Carlo sampling approach enabled us to elucidate cost statistics in random genetic

codes, including a vast number of suboptimal codes. In this study, we also employed a genetic algorithm (GA) simulation to ascertain plausible evolutionary pathways of the genetic code toward optimal and suboptimal codes (Fig. 3). Comparing the GA trajectories and the fitness landscape constructed through Monte Carlo sampling revealed significant differences in the distribution of low-cost codes (Fig. 2a and Fig. 3a), which provide insights into the fitness structure of high-cost codes (e.g., Fig. 3b). Our study has successfully revealed the characteristics of genetic code evolution by integrating optimization and random sampling approaches. To clarify this point, we have revised the manuscript as follows.

Revision #3-3 (Page 9, Line 284):
We also performed GA simulations to analyze possible evolutionary trajectories toward these peaks. Comparing the GA trajectories and the fitness landscape constructed through Monte Carlo sampling revealed significant differences in the distribution of low-cost codes (Fig. 2a and Fig. 3a), which represents that the evolutionary path-dependent bias in the GA arises under the multi-peaked fitness landscape (e.g., Fig. 3b). Specifically, the fraction of independently evolved genetic codes reaching each peak is determined by the basin volume rather than the phase-space volume of these optima. This finding demonstrates the importance of integrating sampling-based and optimization-based approaches.

Comment #3-4: The equation 1 and associated description in Introduction should be moved to Methods.

Response #3-4: According to the reviewer's comment, we have revised the method section.

Comment #3-5: The assumption on selective values of substitutions in the equation 2 was derived from old studies from 1964 and maybe not valid.

Response #3-5: As the reviewer pointed out, our assumption of P(c|c') in Eq. 2 is based on the old experiment and might not be valid. Although this point needs to be improved, the estimation of the mistranslation rate is still difficult even with the recent technology. Thus, most theoretical works (e.g., references [3–6,15]) on the genetic code have been

still based on this old data. Nevertheless, examining other conditions needs to be addressed to validate our claims. To this end, we tested how our results would change with a cost function that reflects experimental data (see below Response #3-6).

Comment #3-6: I think that assumption on at least two acidic amino acids (Asp and Glu) and terminal translation signal is also arbitrary. Other assumptions can change the results.

Response #3-6: As the reviewer pointed out, the restriction enforcing two Asp and Glu does not naturally arises from arguments of the code robustness and is rather artificial. We hypothesized that this condition would be present as a selection pressure separate from the robustness of the code since, without these conditions, the structure of a random code with a cost comparable to the SGC would be far from the current configuration of the SGC. We also confirmed that qualitatively similar results including appearance of the four fitness peaks are obtained without this condition (Fig.S5). To clarify this point, we rewrote the result section at L.111 in the revised manuscript.

Furthermore, to demonstrate the generality of our conclusions, we performed additional simulations using two different cost (fitness) functions: (1) a cost function that includes the amino acid frequencies in the genomes of extant organisms, which mimics the frequencies in proteomes, and (2) a cost function that incorporates an empirical, evolution-based scoring function for amino acid substitution cost (BLOSUM62 score). In both cases, we confirmed that the qualitatively same results to the original cost function are obtained. We added descriptions about these additional simulations at L.111 and the supplementary text with Figs. S6 and S7.

Revision #3-6 (Page 5, Line 111):

So far, we considered an ensemble containing at least two Asp and two Glu (i.e., condition (4) in the Methods section). By performing additional simulations, we also confirmed that our results do not depend on this condition. A closely similar fitness landscape with four peaks appears even in the absence of the condition (see Supplemental text and Fig.S5). Further, we performed simulations with two different cost (fitness) functions: (1) the cost including amino acid frequencies in the genome in extant organisms, and (2) the cost incorporating an empirical evolution-based scoring function for amino acid substitution (BLOSUM62 score~¥cite{henikoff1992amino}). In both cases, similar fitness landscapes with multi-peaks were confirmed. In cost incorporating

BLOSUM score, the orange cluster in the original model (Fig.2: the cluster (d)) that codes high polar requirement amino acids at the fourth column is split into two clusters (d) and (f) under this cost function (Fig.S7a; the orange and purple clusters). However, the amino acid configuration for each peak is similar to the original model (Fig.2c-f).

Comment #3-7: Explain deltaPR in the legend of Fig. 3.

Response #3-7: We have corrected it.

Comment #3-8: The sentence:" Despite their extremely low probability, the number of such codes was ~ 10^59." is repeated.

Response #3-8: We have corrected it.

Comment #3-9: I did not find the detailed description of genetic algorithms in Methods.

Response #3-9: The explanations of GA algorithm were in the results section in the previous manuscript. We have moved the explanations to the Methods section (L.166).

Revision #3-9 (Page 6, Line 166):

We also performed a simple genetic algorithm with population size $N=100$.
Initially, randomly generated N genetic code that satisfies the above (1)-(4) conditions are prepared. In each generation, the top 50¥% of the population of genetic codes was selected according to the cost in Eq.~(¥ref{eq:cost}) and copies of the selected population are generated by random sampling without replacement until the total population reaches $N=100$. In these copies, mutations in the genetic code (i.e., from $a(c)$ to another amino acid, $a'(c)$) occur with a mutation rate $¥mu=0.1$ in each element of $¥ve{a}$. The recombination (crossover) process~¥cite{blazej2016role,blazej2018optimization,santos2011simulated,wnketrzak2018optimality} is not incorporated. When the mutated code does not contain 20 different amino acids, or at least two instances of Asp and Glu, the mutation process starts over. We performed 10^5 independent GA runs. We performed 10^5 independent GA runs with 500 generations and analyzed the statistics of the trajectories.
