# Supplementary material for: Rare-event sampling analysis uncovers the fitness landscape of the genetic code
Source: PLoS Comput Biol. 2023 Apr 17;19(4):e1011034. doi: 10.1371/journal.pcbi.1011034 (PMC10138212; doi:10.1371/journal.pcbi.1011034)
Supplement: Attachment — Submitted filename: Review_PCOMPBIOL-D-22-01373.docx [file pcbi.1011034.s010.docx]

In this manuscript, Omachi et al. revisited a classical question about the robustness of the standard genetic code (SGC) against translational errors. They used a similar framework as previous studies [1-3] to compare the robustness of the SGC to alternative genetic codes (AGCs). Based on the polar requirement (PR) changes by mistranslation, they defined a cost function to evaluate the robustness of genetic codes. Unlike previous studies, Omachi et al. explored a much broader space of AGCs (10^79^) using multicanonical Monte Carlo sampling method. They concluded that one in every 10^20^ AGCs is more robust than the SGC. Using PCA on the PR scale of the code space, they found four major global peaks with similar or higher robustness than the SGC. Applying genetic algorithm analysis, they found the fraction of evolutionary trajectories ending up in each peak is not determined by the phase-space volume of the peaks but by the fraction of initial genotypes that could evolve into each peak. Below, I evaluate their three major findings individually.

The conclusion that the SGC is more robust than every 10^20^ AGCs is interesting. On the one hand, the discrepancy between this conclusion and the classical “one in a million” claim [1] is due to the fact that Omachi et al. broke the block structure of genetic code when simulating AGCs, which naturally makes AGCs less robust than AGCs generated in previous studies. On the other hand, given the 10^79^ genotype space they explored, there will be ~10^59^ AGCs that are at least as robust as the SGC, which is very surprising.

Does the claim about four major fitness peaks provide us valuable information about the evolutionary origin of the SGC? Probably not yet.

(1) The PCA is performed on a subset of AGCs with similar or lower costs than the SGC. If the PCA is performed on all the genotypes, the clustering pattern could be very different. It is unclear why the authors only use this subset to do the dimensionality reduction.
(2) The first two PCs only explained a relatively small proportion of the variance (~40%). Will including more PCs change the clustering pattern? In other words, there are no justifications and power analysis for just using the first two PCs to explore the connectivity of the peaks in the fitness landscape of genetic codes.
(3) Given that there are four global peaks existing, why the SGC ends up in this particular peak? Is it just historical contingency or is it because of selection on other aspects of the SGC?
(4) Since many more genotypes are better than the SGC, why did SGC stop evolution towards a higher peak?
(5) Although the authors mentioned in their discussion that incorporating other parameters, such as codon usage is beyond the scope of this study, the importance and generosity of the findings in the fitness landscape will be significantly impaired without examining it under other conditions. For example, they could use different mistranslation error rates for each codon based on proteomics data, using different amino acid properties to calculate the cost, or considering codon usage [4].

The third point they made is interesting and general to any kind of fitness landscape. This point is essentially talking about the overall shape of peaks, some peaks could have a large basin but become much narrower when reaching the top.

Overall, I think the first and third points are clear. However, there are unclear issues about point 2 and other questions that should be explained and justified before acceptance for publication.

Other questions:

(1) For Fig. 2a, why only AGCs with a cost similar to the SGC are plotted while the PCA and clustering is performed using sample data with a cost similar or lower than the SGC?
(2) There is a sentence repetition in line 240.

Reference:

1.	Freeland SJ, Hurst LD. The genetic code is one in a million. J Mol Evol. 1998;47(3):238-48. Epub 1998/09/11. doi: 10.1007/pl00006381. PubMed PMID: 9732450.

2.	Haig D, Hurst LD. A quantitative measure of error minimization in the genetic code. J Mol Evol. 1991;33(5):412-7. doi: 10.1007/BF02103132. PubMed PMID: 1960738.

3.	Novozhilov AS, Wolf YI, Koonin EV. Evolution of the genetic code: partial optimization of a random code for robustness to translation error in a rugged fitness landscape. Biol Direct. 2007;2:24. Epub 20071023. doi: 10.1186/1745-6150-2-24. PubMed PMID: 17956616; PubMed Central PMCID: PMCPMC2211284.

4.	Bartonek L, Braun D, Zagrovic B. Frameshifting preserves key physicochemical properties of proteins. Proc Natl Acad Sci U S A. 2020;117(11):5907-12. Epub 20200303. doi: 10.1073/pnas.1911203117. PubMed PMID: 32127487; PubMed Central PMCID: PMCPMC7084103.
